# Current situation of endemic mycosis in the Americas and the Caribbean: Proceedings of the first international meeting on endemic mycoses of the Americas (IMEMA)

**DOI:** 10.1111/myc.13510

**Published:** 2022-08-16

**Authors:** Diego H. Caceres, Laura C. Echeverri Tirado, Alexandro Bonifaz, Antoine Adenis, Beatriz L. Gomez, Claudia Lizette Banda Flores, Cristina E. Canteros, Daniel Wagner Santos, Eduardo Arathoon, Elia Ramirez Soto, Flavio Queiroz‐Telles, Ilan S. Schwartz, Jeannete Zurita, Lisandra Serra Damasceno, Nataly Garcia, Norma B. Fernandez, Omayra Chincha, Patricia Araujo, Ricardo Rabagliati, Tom Chiller, Gustavo Giusiano

**Affiliations:** ^1^ Centers for Disease Control and Prevention (CDC) Atlanta Georgia USA; ^2^ Center of Expertise in Mycology Radboudumc/CWZ Nijmegen The Netherlands; ^3^ Studies in Translational Microbiology and Emerging Diseases (MICROS) Research Group, School of Medicine and Health Sciences Universidad del Rosario Bogota Colombia; ^4^ Medical Mycology Group, School of Medicine, Microbiology and Parasitology Department Universidad de Antioquia Medellín Antioquia Colombia; ^5^ Hospital General de Mexico Dr Eduardo Liceaga Ciudad de Mexico Mexico; ^6^ Centre d'Investigation Clinique Antilles Guyane Inserm 1424 Cayenne France; ^7^ Centre Hospitalier de Cayenne Cayenne France; ^8^ Universidad Peruana Cayetano Heredia (UPCH), Hospital Cayetano Heredia Lima Peru; ^9^ Departamento de Micología, Instituto Nacional de Enfermedades Infecciosas (INEI) Administración Nacional de Laboratorios e Institutos de Salud (ANLIS) "Dr. Carlos G. Malbrán" Buenos Aires Argentina; ^10^ Department of Infectious Diseases and Infection Control Universidade Federal do Maranhão, UFMA São Luís Maranhão Brazil; ^11^ Instituto D'Or de Pesquisa e Ensino, IDOR, Hospital UDI São Luis Maranhão Brazil; ^12^ Asociación de Salud Integral Guatemala City Guatemala; ^13^ Centro Nacional de Enfermedades Tropicales (CENETROP) Santa Cruz Bolivia; ^14^ Department of Public Health Federal University of Paraná Brazil; ^15^ Department of Medicine, Division of Infectious Diseases University of Alberta Edmonton Alberta Canada; ^16^ Facultad de Medicina Pontificia Universidad Católica del Ecuador Quito Ecuador; ^17^ Unidad de Investigaciones en Biomedicina, Zurita & Zurita Laboratorios Quito Ecuador; ^18^ Faculdade de Medicina Universidade Federal do Ceará Fortaleza CE Brazil; ^19^ Hospital São José de Doenças Infecciosas, Secretaria de Saúde Ceará Brazil; ^20^ Sociedad Venezolana de Microbiología Caracas Venezuela; ^21^ Sección Micologia, Division Infectología Hospital de Clinicas “José de San Martín” Universidad de Buenos Aires Buenos Aires Argentina; ^22^ Departamento de Bacteriología y Micología Laboratorio Central de Salud Pública, Ministerio de Salud Pública y Bienestar Social Asunción Paraguay; ^23^ Departamento de Enfermedades Infecciosas del Adulto, Escuela de Medicina Pontificia Universidad Católica de Chile Santiago Chile; ^24^ Instituto de Medicina Regional Universidad Nacional del Nordeste, Consejo Nacional de Investigaciones Científicas y Técnicas de Argentina (CONICET) Resistencia Chaco Argentina

**Keywords:** *blastomyces*, *coccidioides*, *histoplasma*, *paracoccidioides*

## Abstract

**Background:**

The Americas are home to biologically and clinically diverse endemic fungi, including *Blastomyces, Coccidioides*, *Emergomyces*, *Histoplasma, Paracoccidioides* and *Sporothrix*. In endemic areas with high risk of infection, these fungal pathogens represent an important public health problem.

**Objectives:**

This report aims to summarise the main findings of the regional analysis carried out on the status of the endemic mycoses of the Americas, done at the first International Meeting on Endemic Mycoses of the Americas (IMEMA).

**Methods:**

A regional analysis for the Americas was done, the 27 territories were grouped into nine regions. A SWOT analysis was done.

**Results:**

All territories reported availability of microscopy. Seventy percent of territories reported antibody testing, 67% of territories reported availability of *Histoplasma* antigen testing. None of the territories reported the use of (1–3)‐β‐d‐glucan. Fifty two percent of territories reported the availability of PCR testing in reference centres (mostly for histoplasmosis). Most of the territories reported access to medications such as trimethoprim‐sulfamethoxazole, itraconazole, voriconazole and amphotericin B (AMB) deoxycholate. Many countries had limited access to liposomal formulation of AMB and newer azoles, such as posaconazole and isavuconazole. Surveillance of these fungal diseases was minimal.

**Conclusions:**

A consensus emerged among meeting participants, this group concluded that endemic mycoses are neglected diseases, and due to their severity and lack of resources, the improvement of diagnosis, treatment and surveillance is needed.

## 1 INTRODUCTION

The Americas contain a diverse group of endemic fungi,^1^, ^2^, ^3^, ^4^ which include the dimorphic fungal pathogens in the genera *Blastomyces*, *Coccidioides*, *Emergomyces*, *Histoplasma* and *Paracoccidioides*; from the order Onygenales (Eurotiomycetes, Ascomycota) and *Sporothrix* from the order Ophiostomatales. Most of these fungi are notable for the ability to cause disease in people with weakened immune systems as well as healthy individuals.^5^ Due to their strong relationship with the environment these fungi are classified as sapronotic disease agents and are able to cause communicable diseases classified as sapronoses in humans, where the source of infection is an abiotic substrate, and the interhuman transmission is unusual.^1^, ^2^, ^3^ These thermally dimorphic fungal pathogens have a filamentous and a saprophytic phase; they are able to grow as a mould in the environment (20–25°C), and as yeast, or spherule in the case of *Coccidioides*, within a mammalian host (37°C).^1^, ^2^, ^3^ Finally, endemic areas of these pathogens should be reviewed due to the expansion of urban territories and a wide range of changes in the environment, including climate change. An increase in endemic fungal disease case reports, even in areas not previously considered endemic, has been observed and has led to an increase in awareness for these diseases. Other factors such as increased use of immunosuppressive agents and even dynamics in human migration are redefining the role of fungal pathogens around the world.^6^

In this context, the first International Meeting on Endemic Mycoses of the Americas (IMEMA) was envisioned to facilitate a comprehensive scientific forum to discuss the systemic endemic mycoses of the Americas. Originally planned for 2020 as an in‐person meeting in Santiago del Estero, a location symbolically chosen due to the sympatric presence of three endemic mycoses in this Argentinian province, the meeting was converted to a virtual forum because of the COVID‐19 pandemic. It was held on two separate days, 29 May and 5 June, 2021. The meeting was supported by governmental, non‐governmental and academic societies. It was divided into a total of 14 sections that included 55 speakers/section coordinators from 12 countries and was attended by a total of 259 participants from 26 countries, mostly from the Americas and the Caribbean region. Participants had the opportunity to interact with region public health authorities, partners, community collaborators and scientists from different American countries. The following topics were discussed during the meeting: epidemiology overview, advances and research findings on pathogenesis, the omics of dimorphic fungi that cause systemic endemic mycoses, trends in treatments and diagnostic developments.

The goal of this report is to summarise the principal findings of the first International Meeting on Endemic Mycoses of the Americas (IMEMA).

## 2 METHODS

Country experiences were grouped into nine regions: (1) the United States and Canada; (2) Mexico, subdivided into three regions (North, Central and South regions); (3) Central America (Belize, Costa Rica, El Salvador, Guatemala, Honduras, Nicaragua and Panama) and The Greater Antilles (Cuba, Hispaniola: Haïti and Dominican Republic, Puerto Rico, Jamaica and the Cayman Islands); (4) The Guiana Shield (French Guiana, Guyana and Surinam) and lesser Antilles (divided into eight independent nations, and numerous dependent and non‐sovereign states, which are politically associated with the United Kingdom, France, the Netherlands and the United States); (5) North Andes (Colombia, Ecuador, Peru and Venezuela); (6) North Brazil; (7) South Brazil; (8) Bolivia, Paraguay and Northern Argentina and (9) Chile, Uruguay and Central and Southern Argentina. Representatives from each of these regions were selected and used a standardised instrument to collect data on disease epidemiology including, outbreak reports, the number of people who are at increased risk for fungal diseases, the availability of antifungals and laboratory assays for routine diagnosis, surveillance and ongoing research.

In addition, a regional Strength, Weakness, Opportunities and Threats (SWOT) analysis was done. Selected regional representatives used this instrument to identify regional strengths, weaknesses, opportunities and threats with the objective to identify needs and future directions for research. For each subject, representatives described the main topics related to (i) access to diagnostic tests; (ii) access to antifungals; (iii) surveillance and (iv) research. Three weeks before to the meeting, a Google Form online assessment, focus on regional weakness and threats, was shared with meeting participants (Supplementary material S1). This assessment covered 35 key topics distributed across the four subjects described above. Assessment participants prioritised weaknesses and threats (from main to low priority). The collection of responses was closed two days before to the first day of the meeting (27 May, 2022). The results of the regional SWOT analysis were presented and discussed by the participants during the first day of the IMEMA meeting (https://www.youtube.com/watch?v=bKA7o8LOsqc).

## 3 RESULTS: STATUS OF THE ENDEMIC MYCOSES IN THE AMERICAS

The nine regions cover a total of 27 territories (Table 1). All territories reported availability of microscopy examination and fungal culture in at least one laboratory service for routine patient diagnosis. Antibody (Ab) testing was reported in 19 of the 27 (70%) territories. Some laboratories use commercial antigen for Ab detection, but in several countries, it is produced in their own laboratories. *Histoplasma* Ab testing was the most reported. French Guiana and Overseas France territories in the Lesser Antilles refer specimens for fungal Ab testing to mainland France. Antigen (Ag) testing was only available for the detection of *Histoplasma* Ag in 18 of the 27 territories (67%). None of the territories reported the use of (1–3)‐β‐D‐glucan (BG) test as a routine assay for the diagnosis of endemic mycoses. Finally, 14 of 27 (52%) territories reported the availability of PCR testing in reference centres, mostly used for the diagnosis of histoplasmosis. Some territories in the Lesser Antilles (Overseas France) reported access to molecular testing only by reference to mainland France (Table 1).

**TABLE 1:** 1st International Meeting on Endemic Mycoses of the Americas (IMEMA): regional analysis on diagnostics, treatment access and disease surveillance

Region/Country	Availability of diagnostics^a^					Availability treatment						Reportable
	Microscopy	Culture	Ab	Ag	Molecular	Sulfas	ITZ	VCZ	D‐B Amp	Lip B Amp	Others	
North America												
Canada	Yes	B, C, H, P	B, C, H, P	B, C, H	B, C, H, P	N/A	Yes	Yes	Yes	Yes	PCZ, IVZ	B, H
Mexico	Yes	B, C, H, P	B, C, H, P	B, C, H	B, C, H, P	Yes	Yes	Yes	Yes	Yes	N/A	No
United States	Yes	B, C, H, P	B, C, H, P	B, C, H	B, C, H, P	N/A	Yes	Yes	Yes	Yes	PCZ, IVZ	B, C, H
Central America												
Belize	Yes	B, H, P	No	H	No	Yes	Yes	Yes	Yes	Yes	KTZ	No
Costa Rica	Yes	B, H, P	H	H	No	Yes	Yes	Yes	Yes	No	KTZ	No
El Salvador	Yes	B, H, P	No	H	No	Yes	Yes	Yes	Yes	Yes	KTZ	No
Guatemala	Yes	B, H, P	H	H	H	Yes	Yes	Yes	Yes	Yes	KTZ	C, H, P
Honduras	Yes	B, H, P	No	H	No	Yes	Yes	Yes	Yes	No	KTZ	No
Nicaragua	Yes	B, H, P	No	H	No	Yes	Yes	Yes	Yes	Yes	KTZ	H
Panama	Yes	B, H, P	H	H	No	Yes	Yes	Yes	Yes	Yes	KTZ	No
North Andes												
Colombia	Yes	H, P	H, P	H	B, C, H, P	Yes	Yes	Yes	Yes	Yes	FCZ, PCZ	No
Venezuela	Yes	B, C, H, P	C, H, P	No	H	Yes	Variable	Variable	Variable	No	FCZ, PCZ	No
Ecuador	Yes	H, P	H, P	H	H	Yes	Yes	Yes	Yes	No	FCZ	No
Peru	Yes	H, P	H, P	H	H	Yes	Yes	Variable	Yes	Variable	FCZ	No
The Guiana Shield												
Guyana	Yes	H	No	No	No	Yes	Yes	No	No	No	No	No
Surinam	Yes	H	No	No	No	Yes	Yes	No	Yes	No	No	No
French Guiana	Yes	H, P	H^b^	H	H	Yes	Yes	Yes	Yes	Yes	FCZ, 5‐FC	No
Brazil (regions)												
North	Yes	C, H, P	No	No	No	Yes	Yes	No	No	Yes	No	No
South	Yes	H, P	H, P	No	H, P	Yes	Yes	No	Yes	Yes	No	No
Bolivia, Paraguay and North Argentina												
Bolivia	Yes	C, H, P	C, H, P	H	No	Yes	Yes	Yes	Yes	Yes	No	No
Paraguay	Yes	C, H, P	C, P	H	No	Yes	Yes	Yes	Yes	Yes	No	No
North Argentina	Yes	C, H, P	C, H, P	No	H, P	Yes	Yes	Yes	Yes	Yes	No	No
Chile, Uruguay and South Argentina												
Chile	Yes	C, H, P	No	No	No	Yes	Yes	Yes	Yes	Yes	PCZ, IVZ, FCZ	No
Uruguay	Yes	C, H, P	C, H, P	No	H	Yes	Yes	Yes	Yes	Yes	PCZ, IVZ, FCZ	No
S outh Argentina	Yes	C, H, P	C, H, P	H	C, H, P	Yes	Yes	Yes	Yes	Yes	PCZ, IVZ, FCZ	C^b^
The Caribbean												
The Greater Antilles	Yes	H	H in Cuba	No	No	Yes	Yes	Yes	Yes	Yes	KTZ	H in Cuba
Lesser Antilles	Yes	H, P	H^b^	No	H^b^	Yes	Yes	Yes	Yes	Yes	FCZ, 5‐FC	No

*Note*: Microscopy also covers histopathology.

*Note*: Catamarca Province Law 5.523. http://www.saij.gob.ar/5523‐local‐catamarca‐programa‐provincial‐prevencion‐deteccion‐tratamiento‐coccidioidomicosis‐lpk0005523‐2017‐11‐23/123456789‐0abc‐defg‐325‐5000kvorpyel?utm_source=newsletter‐mensual&utm_medium=email&utm_term=mensual&utm_campaign=ley‐provincial.

*Note*: The Greater Antilles: Cuba, Hispaniola, Puerto Rico, Jamaica and the Cayman Islands; Lesser Antilles: divided into eight independent nations and numerous dependent and non‐sovereign states (which are politically associated with the United Kingdom, France, the Netherlands and the United States). Over one‐third of the total area and population of the Lesser Antilles lies within Trinidad and Tobago, a sovereign nation comprising the two southernmost islands of the Windward Island chain.

*Note*: Mycoses initials: (B) Blastomycosis, (C) Coccidioidomycosis, (H) Histoplasmosis, (P) Paracoccidioidomycosis.

Abbreviations: 5‐FC, 5‐flucytosine; D‐B Amp, deoxycholate amphotericin B; FCZ, fluconazole; ITZ, itraconazole; IVZ, isavuconazole; KTZ, Ketoconazole; Lip B Amp, liposomal Amphotericin B; N/A, not recommended for treatment; PCZ, Posaconazole; Sulfas, sulfonamide; VCZ, voriconazole.

^a^ Available at least one laboratory service for routine patient diagnosis.

^b^ Specimens tested in France Mainland.

Most of the territories reported access to medications such as trimethoprim‐sulfamethoxazole, itraconazole, voriconazole and amphotericin B (AMB) deoxycholate, with some exceptions including the United States and Canada, where sulfas are not recommended for treating these mycoses. Although the liposomal formulation of AMB is preferred, the availability was limited. In Peru and Venezuela, drug availability was variable. Many countries had limited drug access, with availability only in reference centres. Access to newer azoles, such as posaconazole and isavuconazole, was limited (Table 1).

Surveillance of these fungal diseases was minimal. Systemic endemic mycoses were infrequently mandatorily reportable by public health policies. Only two provinces in Canada report blastomycosis, and one province, histoplasmosis. In the United States, five states report blastomycosis, 27 report coccidioidomycosis and 13 report histoplasmosis. In Guatemala, coccidioidomycosis, histoplasmosis and paracoccidioidomycosis are reportable by FUNGIRED. In Nicaragua, histoplasmosis is a reportable disease. And recently, in Argentina, Catamarca Province added coccidioidomycosis as a reportable disease (Law 5.523, San Fernando Del Valle De Catamarca, Argentina. November 23rd, 2017) (Table 1).

**FIGURE 1:**
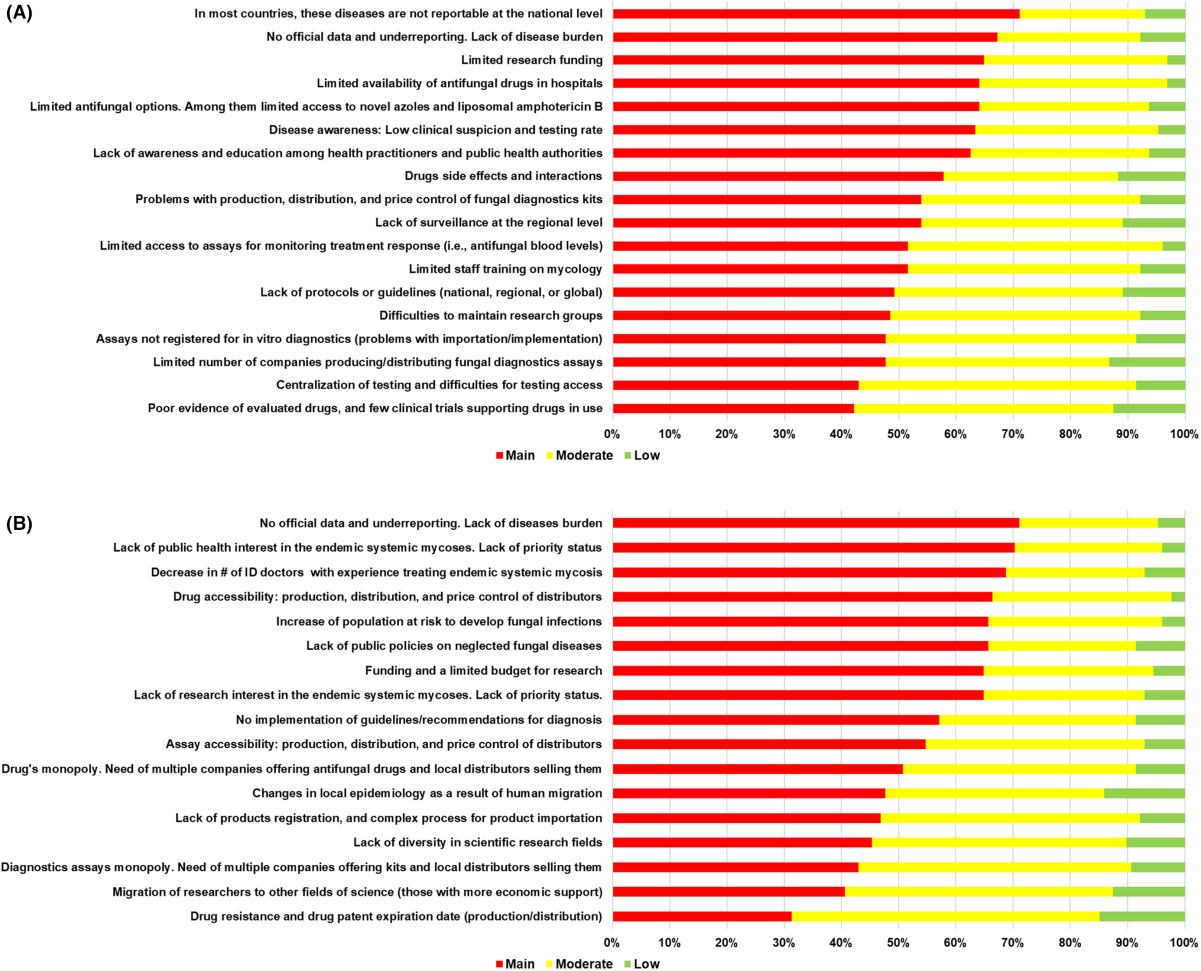
(A) Validation of SWOT analysis Weaknesses. (B) Validation of SWOT analysis Threats.

For the regional SWOT analysis, using the online assessment, we received responses from a total of 128 meeting participants, from 23 countries. On this SWOT, participants indicated as main priorities the need for surveillance of fungal diseases, the limited funding and programme budgets were common concerns. Detailed results of the regional SWOT analysis and validation are summarised in Table 2(A–D) and Figure 1.

**TABLE 2:** Summary of SWOT analysis

(A) Strengths
Diagnosis Most of the regions reported having national reference laboratories (NRL) supporting fungal diagnostics (limitations in Central America, The Greater Antilles countries and Bolivia). In countries with NRL laboratory capacity was diverse Most countries reported experience using conventional testing (microscopy examination and culture) and some expressed the use of Ab testing There are three reference laboratories in the region supporting other countries (CDC, Malbrán and Fiocruz) There is only one company producing commercial kits for fungal diagnostics (producing rapid diagnostic assays) “Manual de infecciones fúngicas sistémicas” by Asociación Panamericana de Infectología (API) Treatment All countries reported availability of main antifungals treatments (fluconazole, itraconazole and amphotericin B‐deoxycholate) The USA and Canada reported substantial experience producing new drugs. In addition, most participant countries reported experience evaluating antifungals Large experience in the treatment of histoplasmosis associated with HIV infection “Manual de infecciones fúngicas sistémicas” by API Surveillance Reportable Fungal Diseases: □ *Blastomycosis (Blasto): USA and Canada (state/province level)* □ *Coccidioidomycosis (VF): USA, Mexico and Argentina (state level). Venezuela NRL report statistics to the Ministry of Health (MOH)* □ *Histoplasmosis (Histo): USA and Canada (state/province level). Cuba and Nicaragua (national level). Venezuela NRL report statistics to the MOH* □ *Paracoccidioidomycosis (PCM): Argentina (surveillance). Brazil is planning to include PCM in the list of reportable diseases. Venezuela NRL report statistics to the MOH* PAHO is promoting activities related to fungal diseases detection CDC MDB, probably the only public health agency with a full‐time dedication of an epidemiology team MDB is promoting the annual fungal diseases awareness week (*#ThinkFungus*), in partnership with other groups such as ISHAM LATAM, GAFFI, MSGERC, ASM, ECMM and national microbiology/mycology societies Research Most countries reported that the research groups were working on a diversity of research topics, among them new diagnostics assays, vaccine development, weather/environmental modelling for prediction of endemic regions (including the effect of environmental changes), development of novel drugs, fungal genomics and fungal epidemiology Recently, countries in Central America are publishing data from the implementation of laboratory capacity mostly focus on HIV care Some countries reported available funding for research and researcher training Long‐standing experience on HIV‐associated histoplasmosis (advocacy, epidemiology and laboratory) Research collaborations and training (clinical and laboratory)
(B) Weaknesses
Diagnosis All participant countries reported centralisation and difficulties for testing □ Need of rapid point‐of‐care testing (POC) for immuno‐diagnosis and molecular detection Difficulties for the transportation of specimens for testing Disease awareness: low clinical suspicion and testing rate Central America, The Greater Antilles and Bolivia: few countries have reference laboratories There is a fundamental need for qualified mycologists, especially outside the main urban areas Need of improved assays for the diagnosis of Blasto and PCM Fungal diagnostics: □ Limited number of companies producing/distributing fungal diagnostics assays □ Problems with production, distribution and price control of fungal diagnostics kits □ Not registered assays for in vitro diagnostics (problems with importation/implementation) Limited access to assays for monitoring treatment response (i.e. antifungal blood levels) Treatment Limited antifungal options □ Most countries reported limited access to novel azoles and liposomal amphotericin B Small sample size in most published clinical trials. Poor evidence of evaluated drugs and few clinical trials supporting the drugs in use Lack of national protocols/PAHO‐WHO guidelines for the management of patients with endemic mycosis Limited availability of antifungal drugs in hospitals Limited knowledge of drugs side effects and interactions Surveillance In most countries, these diseases are not reportable at the national level Lack of awareness and education among health practitioners and public health authorities Lack of surveillance at the regional level Research Most of the countries reported limited research funding Difficulties to maintain research groups Shortage of biorepositories of specimens for new assay development, validation and evaluation Need for new drugs, therapy targets and treatment regimens Limited (lacking) clinical trials for evaluation of new drugs
(C) Opportunities
Diagnosis Novel approaches for rapid testing, using portable and highly accurate technologies WHO essential diagnostics list. Support new test development, production, distribution, registration and price control Most countries of the region could improve their diagnostic capabilities (100% of countries having mycology reference laboratories) Large experience validating and evaluating novel assays Improve fungal suspicion by training and education. A new generation of mycologists is needed □ Taking advantage of novel technologies for face‐to‐face training and e‐learning Improvement of public policies about importation of laboratory reagents to make them available Treatment New clinical trials. Multicentre studies with larger sample sizes New guidelines (national and global) Improve access to telemedicine Improve the treatment regimens: shortest treatment, the second use of approved drugs and co‐infections WHO essential medications. Support new drug development, production, distribution, registration and price control. Promote generic antifungals distribution Improve access to antifungal drugs outside the main urban areas Surveillance Improve capacity to do surveillance. Taking advanced Information technologies (IT) XXI century surveillance Public awareness of fungal diseases. Use of social media and other communication media to promote awareness related to fungal infections Network for surveillance of fungal diseases. Multilayered (hospital, local, state, region, country, WORLDWIDE) Field studies One‐Health (human, animal, vegetal and environmental health) Research Maintain and promote research in diverse areas (epidemiology, diagnosis and treatment) International cooperation. Establish strong international networks of researchers Cooperation with the private sector Promote a new generation of researchers. Scholarships for researcher training Implementation of eco‐epidemiological research work Screening and impact of early diagnosis on mortality Certified bio‐repository collections
(D) Threats
Diagnosis Diagnostics assays monopoly. Need of multiple companies offering kits and local distributors selling them Assay accessibility: production, distribution and price control for distributors Lack of products registration and high complexity process for product importation No implementation of guidelines/recommendations for diagnosis Migration of mycology professionals to other laboratory areas Treatment Drug's monopoly. Need of multiple companies offering antifungal drugs and local distributors selling them Drug accessibility: production, distribution and price control for distributors Drug resistance and drugs patent expiration date (production/distribution) No implementation of guidelines/recommendations for treatment Decrease in the number of doctors specialised in infectious diseases with experience treating endemic systemic mycosis Surveillance Lack of public health interest in the endemic systemic mycoses. Lack of priority status No official data and underreporting. Lack of diseases burden Increase of population at risk to develop fungal infections Changes in local epidemiology as a result of human migration Lack of public policies on neglected fungal diseases Research Migration of researchers to other fields of science (those with more economic support) Funding and a limited budget for research Lack of research interest in the endemic systemic mycoses. Lack of priority status Lack of diversity in scientific research fields

## 4 CONCLUSION

The first IMEMA gathered a multidisciplinary audience of physicians, laboratory professionals, epidemiologists, public health practitioners and representatives from scientific and nonprofit organisations. Diagnostic capability using conventional tools, such as microscopy and culture, was determined to be accessible in the whole region, at least in one reference centre in each territory. The availability of immunological diagnostic methods was uneven throughout in the Americas. Access to non‐standardised Ab testing was reported in about two‐thirds of the regions analysed, and Ag testing, for the diagnosis of histoplasmosis only, was reported in a similar proportion of countries (66%). Molecular testing availability was restricted to half of the territories, in most of these for the diagnosis of histoplasmosis and only in referral centres. We observed that the diagnosis of endemic systemic mycoses has progressed in recent years, but not all mycoses had the same development, with the diagnosis of histoplasmosis having the greatest advances.^7^ Paracoccidioidomycosis, prevalent in Latin America, had the fewest diagnostic tools available, due to the need for an experienced operator.

Access to treatments with sulfonamide, azoles (such as itraconazole and voriconazole) and deoxycholate amphotericin B were reported as available in most territories, but their limited presence in non‐referral hospitals was a major problem in several countries. The lack of national protocols, surveillance and reporting for fungal diseases in most countries of the Americas may contribute to the limited availability of newer drugs such as liposomal AMB and the new generation of azoles, since the disease burden is not quantified and may translate to lack of funding. These issues may also limit research and the development of diagnostic methods for all these endemic diseases.

As important strengths and opportunities, this regional analysis revealed the presence of experts on diagnosis and treatment of these fungal diseases, as well as several organisations that were focused on promoting the advocacy and awareness of these mycoses. Additionally, it is well known that implementation of highly accurate and rapid tests has a significant impact on the detection of the cases and the reduction of mortality. This is well recognised in special for histoplasmosis, related to the implementation of antigen detection testing, medical training and consolidation of laboratory hubs. Despite that, none of these studies previously done were part of governmental programmes, making all these initiatives unviable in the long term, highlighting the importance of the inclusion of fungal diseases in public health programmes, as well as its mandatory reporting, which will be essential for strengthening programmes and burden estimation of these diseases.^8^, ^9^, ^10^, ^11^, ^12^, ^13^, ^14^

The first IMEMA generated a starting point for international cooperation and established a strong international network for training and education of a new generation of mycologists whose research includes eco‐epidemiological investigations and are seeking novel approaches for rapid testing for all systemic mycoses. A consensus emerged among meeting participants, who represented all regions of the Americas, this group concluded that endemic mycoses are neglected diseases, and due to their severity and lack of resources, the improvement of diagnosis, treatment and surveillance is need.

## DISCLAIMER

The findings and the conclusions in this report are those of the authors and do not necessarily represent the official position of the Centers for Disease Control and Prevention.

## Supporting information


Supplementary material S1

## Data Availability

The data that support the findings of this study are available from the corresponding author upon reasonable request.
