# Supplementary material for: Current situation of endemic mycosis in the Americas and the Caribbean: Proceedings of the first international meeting on endemic mycoses of the Americas (IMEMA)
Source: Mycoses. 2022 Aug 16;65(12):1179–87. doi: 10.1111/myc.13510 (PMC9804294; doi:10.1111/myc.13510)
Supplement: Supplementary file 1 — Supplementary material S1 [file MYC-65-1179-s001.pdf]

IMEMA Weaknesses and Threats Validation

Dear participant,

The objective of this survey is to validate the findings of the SWOT (strengths, weaknesses, opportunities, and threats) analysis, focused on the results of the weaknesses and threats identified in the Americas and the Caribbean.

These findings and validation will be presented at the round table of session # 3 of IMEMA, Status of endemic mycoses in the Americas, on Saturday, May 29.

For this, we kindly ask you to take 20 minutes of your time to answer the following survey. Please send your answers by Friday, May 28th, at noon, Buenos Aires time.

* Required

1. Full name *

2. E-mail *

3. Country of residence *

4. City of residence *

IMEMA Weaknesses Validation

5. On a priority scale, how would you classify the following weaknesses: *

Mark only one oval per row.

	Low-priority	Moderate priority	Main priority
**Centralization of testing and difficulties for testing access**			
**Disease awareness: Low clinical suspicion and testing rate**			
**Limited staff training on mycology**			
**Limited number of companies producing/distributing fungal diagnostics assays**			
**Problems with production, distribution, and price control of fungal diagnostics kits**			
**Assays not registered for in vitro diagnostics (problems with importation/implementation)**			
**Limited access to assays for monitoring treatment response (i.e., antifungal blood levels)**			
**Limited antifungal options. Among them limited access to novel azoles and liposomal amphotericin B**			

**Poor evidence of evaluated drugs, and few clinical trials supporting drugs in use**			
**Lack of protocols or guidelines (national, regional, or global)**			
**Limited availability of antifungal drugs in hospitals**			
**Drugs side effects and interactions**			
**In most countries, these diseases are not reportable at the national level**			
**Lack of awareness and education among health practitioners and public health authorities**			
**Lack of surveillance at the regional level**			
**No official data and underreporting. Lack of disease burden**			
**Limited research funding**			
**Difficulties to maintain research groups**			

6. Please, add other weaknesses you may consider relevant not listed in the questionnaire above

IMEMA Threats Validation

7. On a priority scale, how would you classify the following threats: *

Mark only one oval per row.

	Low-priority	Moderate priority	Main priority
**Diagnostics assays monopoly. Need of multiple companies offering kits and local distributors selling them**	⬭	⬭	⬭
**Assay accessibility: production, distribution, and price control of distributors**	⬭	⬭	⬭
**Lack of products registration, and complex process for product importation**	⬭	⬭	⬭
**No implementation of guidelines/recommendations for diagnosis**	⬭	⬭	⬭
**Drug's monopoly. Need of multiple companies offering antifungal drugs and local distributors selling them**	⬭	⬭	⬭
**Drug accessibility: production, distribution, and price control of distributors**	⬭	⬭	⬭
**Drug resistance and drug patent expiration date (production/distribution)**	⬭	⬭	⬭
**Decrease in the number of doctors specialized in**	⬭	⬭	⬭

**infectious diseases with experience treating endemic systemic mycosis**			
**Lack of public health interest in the endemic systemic mycoses. Lack of priority status**	◯	◯	◯
**No official data and underreporting. Lack of diseases burden**	◯	◯	◯
**Increase of population at risk to develop fungal infections**	◯	◯	◯
**Changes in local epidemiology as a result of human migration**	◯	◯	◯
**Lack of public policies on neglected fungal diseases**	◯	◯	◯
**Lack of research interest in the endemic systemic mycoses. Lack of priority status.**	◯	◯	◯
**Lack of diversity in scientific research fields**	◯	◯	◯
**Migration of researchers to other fields of science (those with more economic support)**	◯	◯	◯
**Funding and a limited budget for research**	◯	◯	◯

8. Please, add other threats you may consider relevant not listed in the questionnaire above

This content is neither created nor endorsed by Google.

Google Forms
